# An Unusual Case of Isolated Duodenal Varices With Superior Mesenteric Vein Thrombosis

**DOI:** 10.7759/cureus.43783

**Published:** 2023-08-20

**Authors:** Adbulmalik M Alsabban, Mohammed J Almatrafi, Zaffar M Malik, Inayatulla Y Khiji, Mohammed K Shariff

**Affiliations:** 1 Digestive and Liver Center (DLC), King Abdullah Medical City, Makkah, SAU; 2 Gastroenterology, King Abdullah Medical City, Makkah, SAU; 3 Digestive and Liver Center (DLC) and Advanced Endoscopy Center, King Abdullah Medical City, Makkah, SAU

**Keywords:** upper gastrointestinal(ugi) bleeding, esophagogastroduodenoscopy (egd), endoscopic variceal banding, mesenteric vein thrombosis, duodenal varices

## Abstract

Duodenal varices usually occur due to portal hypertension and are rare causes of gastrointestinal tract bleeding. We report a unique case of a previously fit patient who presented with melena and was found to have isolated duodenal varices (DV) in the third part on esophagogastroduodenoscopy. No esophageal or gastric varices were noticed. The duodenal varices were successfully managed by endoscopic banding. A computerized tomography scan of the abdomen to further investigate the cause confirmed duodenal varices and revealed superior mesenteric vein thrombosis. The liver was normal with patent hepatic and portal veins. No evidence of thrombophilia was found. Apixaban was prescribed for superior mesenteric vein thrombosis and on follow-up. no further bleeding was reported.

## Introduction

Duodenal varices (DV) are an uncommon cause of gastrointestinal tract bleeding (GITB) usually associated with portal hypertension. Isolated DV due to non-portal hypertension is rare and is limited to case reports. Here, we report a case with no past history of cirrhosis, portal hypertension, or coagulopathy disorder that presented with duodenal varices resulting in melena due to superior mesenteric vein thrombosis.

## Case presentation

**Figure 1:**
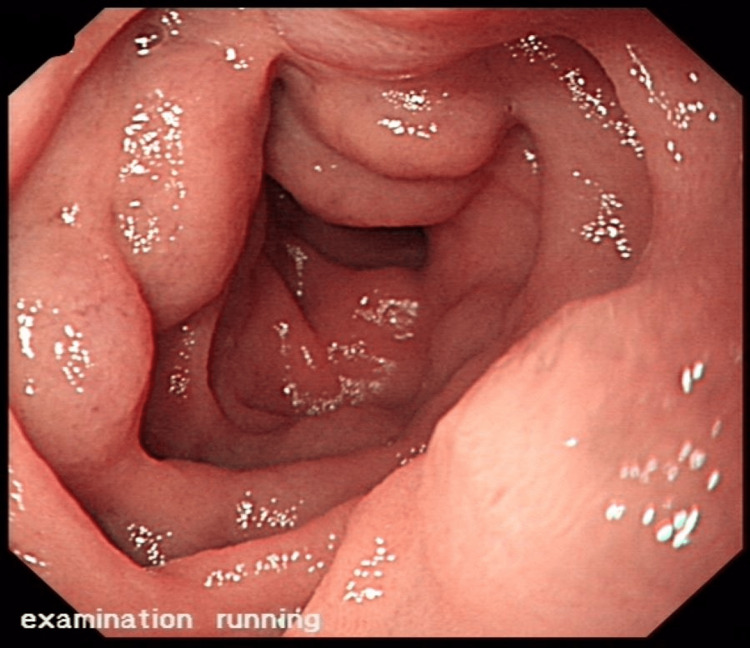
Multiple large varices in the third part of the duodenum

A 35-year-old male, previously fit, presented to the emergency department with a history of melena and a significant drop in hemoglobin levels to 6.2 gm/dl. He was hemodynamically stable, and the general physical examination, including the abdomen, was unremarkable. A digital rectal examination confirmed the presence of melena. Other laboratory investigations, including white cell count, platelet count, lipase, and liver and renal profile were normal. The patient underwent esophagogastroduodenoscopy (EGD) that showed normal esophagus, stomach, and the first two parts of the duodenum without any evidence of varices in the esophagus or stomach or portal hypertensive gastropathy. To carefully examine the upper GI tract and find the cause of GITB, the endoscopist intubated deep into the third part of the duodenum, which surprisingly showed four enlarged varices in the third part (Figure 1).

**Figure 2:**
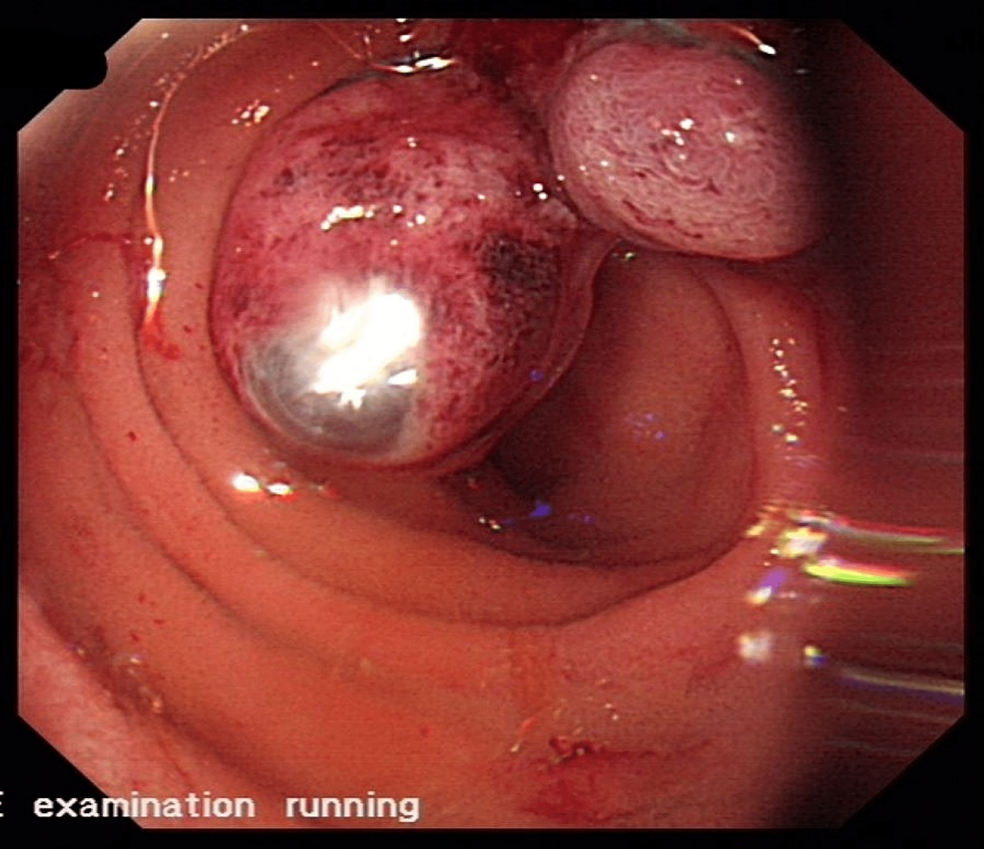
Duodenal varices post banding

Two of the largest duodenal varices were banded successfully (Figure 2).

**Figure 3:**
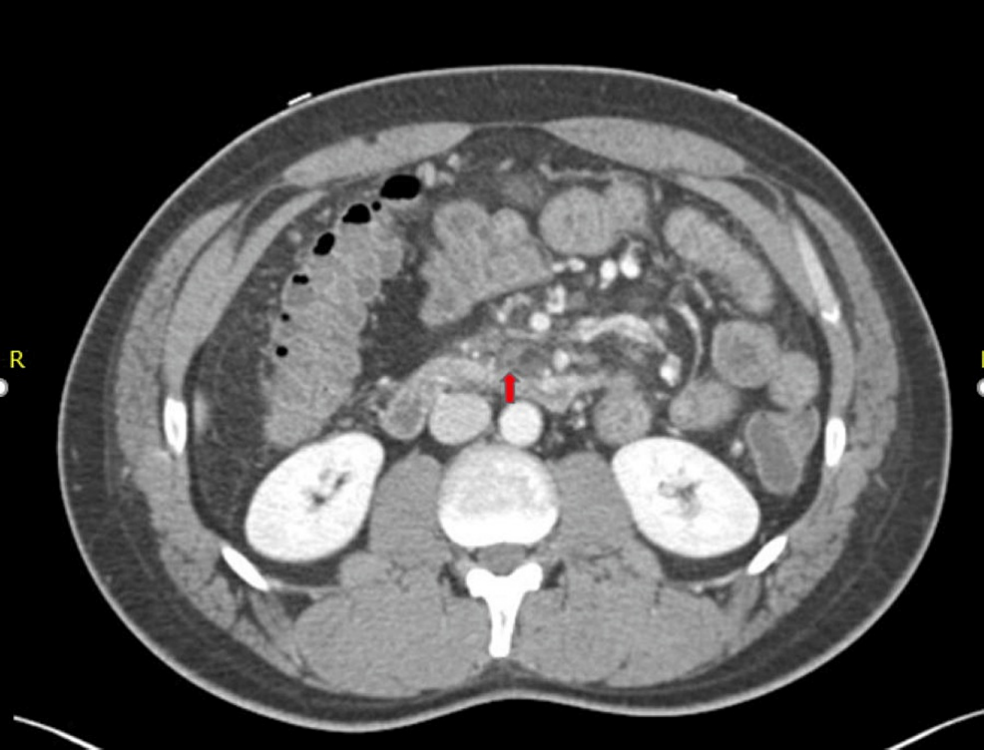
Thrombus in the superior mesenteric vein (red arrow)

**Figure 4:**
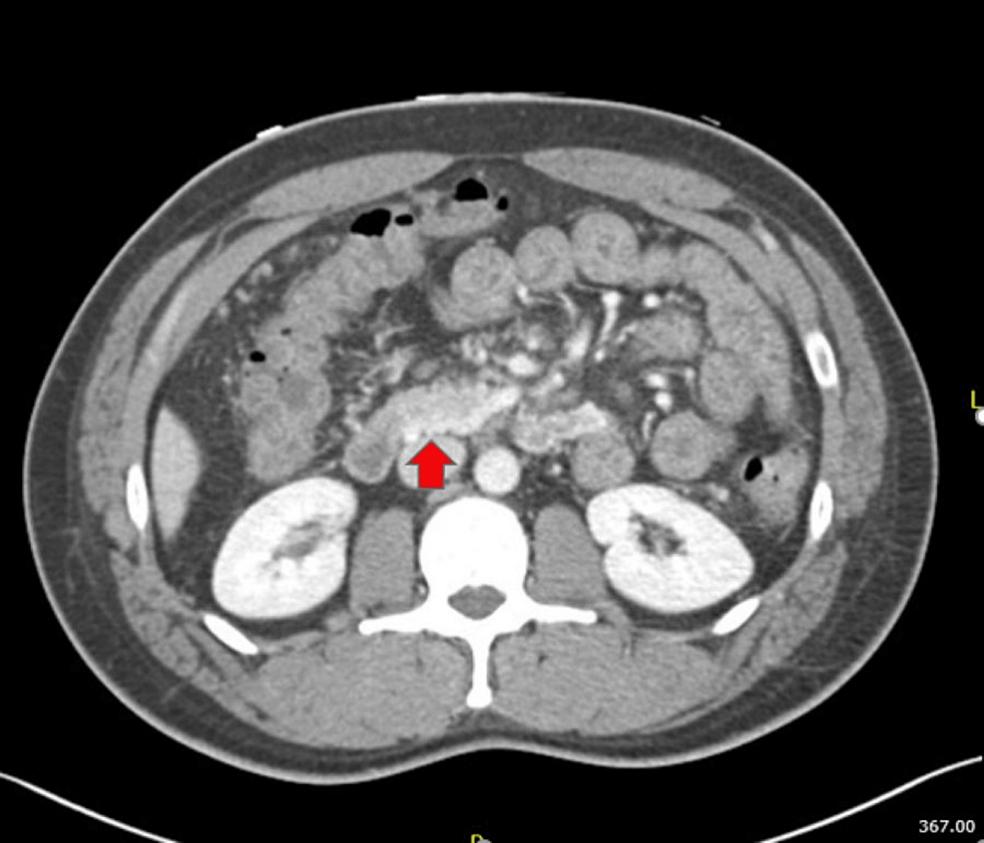
Large duodenal varices (red arrow)

Further investigation by computerized tomography (CT) angiography revealed chronic superior mesenteric vein (SMV) thrombosis (Figure 3), duodenal varices, unremarkable pancreas (Figure 4), and average-sized liver with homogeneous enhancement without any focal lesions. The portal and hepatic veins were patent. A thrombophilia screen to look for the cause of thrombosis like Protein C, S, anti-cardiolipin antibodies, and Factor V Leiden were within the normal range. In addition, vasculitis works up for antinuclear antibody, p- and c-antineutrophil cytoplasmic autoantibody were negative. Following banding, the patient had no more bleeding, and he was started on apixaban for thrombosis. The patient was followed up in the outpatient clinic a few weeks later and had no more melena.

## Discussion

Duodenal varices are part of a heterogeneous group of ectopic varices that arise due to shunting within the portal vascular bed or the systemic/mesenteric vessels [1]. An uncommon manifestation of portal hypertension, DV usually occurs along with oesophageal and/or gastric varices [2,3]. The most common cause of DV is extrahepatic portal hypertension followed by cirrhosis. Isolated DV is unusual and in the context of non-portal hypertension, rarely seen [4]. SMV thrombosis due to various etiologies like coagulopathy, pancreatitis, trauma, or vascular anomalies has been cited mainly in a few case reports as one of the non-portal hypertensive pathologies that cause DV [4-6].

Prompt diagnosis and therapy of DV are essential to avoid massive bleeding and consequential high mortality [5,7]. EGD is the primary modality for diagnosis and therapy. However, detecting DV may be challenging especially if isolated. The DV bulge may be subtle and mimic enlarged mucosal folds or its location in the third or fourth part of the duodenum may be beyond the reach of a routine EGD examination [7,8]. As exemplified in our case the isolated DV was located in the third part of the duodenum and was detected incidentally on careful endoscopic inspection by an astute endoscopist. This emphasizes the importance of raising awareness of DV as a potential cause of GI bleed. The second line of investigation for DV is CT of the abdomen with contrast or CT angiogram that can both detect and treat DV.

Therapeutic management of DV is not standardized due to its uncommon occurrence and treatment is mainly based on the outcomes of case series and case reports. Endoscopic therapy for DV is reported to be effective and options include endoscopic banding, cyanoacrylate injection, thrombin injection, and hemoclip placement, with no clear benefit of one over the other [7,9]. Hence, the choice of therapy is dictated by locally available skills and equipment. Nevertheless, the duodenal anatomy of a thin wall with a torturous and narrow lumen demands good endoscopic skills for safe and successful therapy [10]. The choice of endoscopic banding in our case was influenced by the endoscopist's experience and preference. In cases that rebleed following endoscopic therapy, interventional radiology may offer salvage or definitive therapy in the form of a transjugular intrahepatic portosystemic shunt or balloon occluded retrograde transvenous obliteration of the varices [7,9]. The mainstay of treatment of SMV is anticoagulation and the duration varies from three to six months in cases of reversible etiology. However, in patients like our case without an identifiable cause, long-term anticoagulation is advocated.

## Conclusions

Our case highlights the importance of being aware of duodenal varices that may exist in isolation. In addition, it demonstrates the significance of inspecting the duodenum beyond D2, especially in upper GI bleeding cases with no apparent identifiable source. Although endoscopic therapy for DV may seem daunting, it nevertheless is feasible and effective.
